# A Comprehensive Diagnosis Method of Rolling Bearing Fault Based on CEEMDAN-DFA-Improved Wavelet Threshold Function and QPSO-MPE-SVM

**DOI:** 10.3390/e23091142

**Published:** 2021-08-31

**Authors:** Yi Wang, Chuannuo Xu, Yu Wang, Xuezhen Cheng

**Affiliations:** College of Electrical Engineering and Automation, Shandong University of Science and Technology, Qingdao 266590, China; skd992631@sdust.edu.cn (Y.W.); xuchuannuo@sdust.edu.cn (C.X.); wangyu007@sdust.edu.cn (Y.W.)

**Keywords:** rolling bearing fault, CEEMDAN, DFA, improved wavelet threshold, QPSO, MPE, SVM

## Abstract

A comprehensive fault diagnosis method of rolling bearing about noise interference, fault feature extraction, and identification was proposed. Based on complete ensemble empirical mode decomposition with adaptive noise (CEEMDAN), detrended fluctuation analysis (DFA), and improved wavelet thresholding, a denoising method of CEEMDAN-DFA-improved wavelet threshold function was presented to reduce the distortion of the noised signal. Based on quantum-behaved particle swarm optimization (QPSO), multiscale permutation entropy (MPE), and support vector machine (SVM), the QPSO-MPE-SVM method was presented to construct the fault-features sets and realize fault identification. Simulation and experimental platform verification showed that the proposed comprehensive diagnosis method not only can better remove the noise interference and maintain the original characteristics of the signal by CEEMDAN-DFA-improved wavelet threshold function, but also overcome overlapping MPE values by the QPSO-optimizing MPE parameters to separate the features of different fault types. The experimental results showed that the fault identification accuracy of the fault diagnosis can reach 95%, which is a great improvement compared with the existing methods.

## 1. Introduction

Rolling bearings are the most important parts of rotating machinery and are widely used in modern mechanized equipment [1,2]. The fault of rolling bearing components can cause serious damage to the running status of the machine and the production process. Therefore, it is important to explore a new fault diagnosis technique. However, in practical engineering, the collected bearing vibration signals contain various interference signals (e.g., white noise, harmonic interference), and have nonlinear and nonstationary properties, which makes it difficult to distinguish the bearing fault types and to identify them [3]. Therefore, to improve the fault diagnosis accuracy of rolling bearings, this paper researched three aspects of signal denoising, fault feature extraction, and fault type identification of rolling bearings.

Common vibration signal denoising methods include empirical mode decomposition (EMD) [4], discrete wavelet transform (DWT) [5], singular-value decomposition (SVD) [6,7,8], and so on. Abdelkader et al. [9], based on EMD algorithm, proposed a method to determine the travel point based on the energy values of the IMF components of each order, and an improved wavelet threshold was used to denoise the selected IMF component-bearing vibration signals. Zhao et al. [10] fused the EMD algorithm and selected and tested (ST) the algorithm to denoise the signal, solving the low accuracy in the low-speed operation of the bearing of the EMD denoising method based on the number of interrelationships and the kurtosis criterion. Gao et al. [11] added the CEEMDAN algorithm to adaptive noise, further reducing the modal effect, yielding better convergence, and realizing the fault diagnosis of rolling bearings.

The hard and soft thresholding functions in wavelet thresholding denoising are widely used, but both functions have their shortcomings and can cause a certain degree of distortion when reconstructing the signal. Tajeddini et al. [12] proposed a generic threshold function based on an unbiased risk estimation method to carry out wavelet-packet transform preprocessing of the vibration signal, followed by generic threshold denoising. Kumar et al. [13] proposed an improved modified kurtosis-mixing threshold rule to denoise the vibration signal, which improved the signal-to-noise ratio. Yang et al. [14] used a particle-swarm algorithm to perform adaptive optimal processing of the initial parameters of the wavelet threshold function, which solved the problem of the initial parameters being influenced by empirical values and overcame the defects such as signal noise removal not being complete enough or removing the useful information. Chegini et al. Ref. [15] proposed a new method of bearing vibration signal denoising based on empirical wavelet transform (EWT) as well as the threshold function, which better overcame the defect of constant deviation of the traditional soft threshold function. However, the denoising performance of the wavelet threshold denoising method is closely related to the wavelet basis function and the threshold function, and is not adaptive. Although the CEEMDAN algorithm is adaptive, the definition of noise and useful signal is relatively vague, resulting in high distortion of the denoised signal. Shi et al. [3] proposed a de-trending fluctuation analysis technique for the critical values of the noise component and the useful signal component. This method ensures the accuracy of signal correlation discrimination to a certain extent. Based on the CEEMDAN algorithm decomposition of the vibration signal, Chaabi et al. [16] used wavelet analysis, principal component analysis, and order tracking analysis to perform multi-method denoising. In order to improve identification accuracy of rolling bearings with nonlinear and nonstationary vibration signals, Chen et al. [17] proposed a novel fault diagnosis method based on wavelet thresholding denoising and CMEEMDAN with adaptive noise. To verify the denoising effectiveness of combined wavelet thresholding and CEEMDAN, Bie et al. [18] added random Gaussian white noise to the bearing fault signal to simulate the actual noise disturbance of rolling bearings, and adopted the CEEMDAN-wavelet threshold function to the denoising method. The experimental results showed that the combined denoising method could effectively remove the interference of noise. Therefore, based on the application characteristics of each denoising method, this paper proposed a denoising method based on a CEEMDAN-DFA-improved wavelet threshold function. The method uses the CEEMDAN algorithm to decompose the vibration signal, performs detrended fluctuation analysis (DFA) on the obtained eigenmode function (IMF), calculates the scalar function value of each IMF component, selects the noise-dominated IMF component, and applies an improved wavelet threshold function to denoise it.

The current methods of fault feature extraction for rolling bearings are mainly time-domain analysis, frequency domain analysis, and time-frequency domain analysis; among them, the time-frequency domain analysis method is most commonly used. Zhen et al. [19] decomposed the vibration signal using wavelet packets and selected a suitable bandwidth according to the Kurtosis spectrum correlation theory, and finally applied the envelope spectrum analysis to extract the eigenfrequencies. Han et al. [20] used the Teager energy operator to enhance the signal after wavelet denoising and then extracted the bearing fault features by the CEEMD algorithm. Li et al. [21] used exchange entropy (PE) for bearing fault feature extraction, but could not measure multi-scale signals. Multi-scale variational entropy (MPE) [22,23] was introduced into fault diagnosis by Yin et al. [24]. Du et al. [25] used MPE to extract fault features and combined them with a self-organizing fuzzy classifier based on the harmonic mean difference (HMDSOF) to classify the fault features. MPE can respond to the changes of vibration signals very well, but its parameters have a great influence on the calculation of entropy values. If the parameters are not selected properly, it will cause the arrangement entropy values of multiple signals of bearings to be mixed, and thus the type of bearing failure cannot be identified. In order to achieve complete separation of arrangement entropy values under different operating conditions of the bearings, this paper adopted quantum particle swarm optimization (QPSO) to optimize the initial parameters of MPE, and then selected the appropriate MPE entropy values to construct the rolling bearing fault feature vector.

Common fault classification and identification methods include artificial neural networks (ANN) [26], extreme learning machines (ELM) [27,28], and support vector machines (SVM) [29,30]. The ANN has made possible many achievements in the field of pattern identification, but its identification performance is strongly influenced by parameters and it is easy to fall into local minima during the optimization process. Although ELM runs fast, its generalization performance is poor. SVM has fewer adjustable parameters and runs stably. It can obtain higher diagnostic accuracy under the condition of fewer training samples [24]. Therefore, this paper used a SVM for fault identification of rolling bearings. This paper also proposed a comprehensive rolling bearing fault feature extraction and identification method based on the combination of QPSO-MPE-SVM.

The main work and contributions of this paper are summarized as follows:

(1) A CEEMDAN-DFA-improved wavelet thresholding denoising method was proposed. The method uses the CEEMDAN algorithm to decompose the vibration signal, performs DFA on the obtained IMF, calculates the scalar function value of each IMF component, selects the noise-dominated IMF component, and applies an improved wavelet threshold function to denoise it.
(2) Combining QPSO-MPE-SVM into an effective fault diagnosis method can accurately extract fault features and improve the identification accuracy of bearing faults.
(3) Experimental cases were used to illustrate the effectiveness of the proposed method in bearing vibration signal denoising, fault feature extraction, and fault identification.

The rest of this paper is organized as follows. The CEEMDAN-DFA-improved wavelet thresholding denoising method is introduced, and its validity is verified in Section 2. The QPSO-MPE-SVM fault feature extraction and diagnosis method is presented, and its validity is verified in Section 3. The effectiveness of the proposed method is proven by experimental example in Section 4. In addition, contrastive analysis among the different methods is conducted. Finally, some conclusions are summarized in Section 5.

## 2. Denoising Algorithm of the CEEMDAN-DFA-Improved Wavelet Threshold Function

### 2.1. Basic Algorithm Related to CEEMDAN-DFA-Improved Wavelet Threshold Function

#### 2.1.1. CEEMDAN Algorithm

To overcome the high computing time and the residue of added noise present in the IMFs, and to better deal with the modal mixing problem of empirical modal decomposition (EMD), the ensemble empirical mode decomposition (EEMD) was proposed by Liu et al. in 2009 [31]. The EEMD algorithm is effective in suppressing modal aliasing, but a certain degree of distortion is produced when the signal is reconstructed. The improved CEEMDAN algorithm was proposed by Colominas et al. in 2014 [32]. The improved CEEMDAN algorithm is summarized as follows.

(1) The *j*th IMF component generated by the signal decomposition by the EMD is defined as $E_{j}\left(\cdot \right)$. The *j*th IMF by the CEEMDAN is defined as $IMF_{j}{}^{\prime }$. $n^{i}\left(t\right)$ is for the Gaussian white noise. The CEEMDAN performs *I* EMD decomposition on the noisy signal $x\left(t\right)+\varepsilon_{0}\cdot n^{i}\left(t\right)$ formed by the combination of the original signal and the white noise. Then the first IMF component decomposed by CEEMDAN can be expressed as:
  (1) $$IMF_{1}^{\prime }\left(t\right)=\frac{1}{I}\sum_{i=1}^{I}IMF_{1}^{i}\left(t\right)$$
(2) First residual sequence of the first stage (*j* = 1) is expressed as:
  (2) $$r_{1}\left(t\right)=x\left(t\right)-IMF_{1}^{\prime }\left(t\right)$$
(3) The $r_{1}\left(t\right)+\varepsilon_{1}E_{1}\left(n^{i}\left(t\right)\right)\left(i=1,2,\cdot \cdot \cdot \right)$ is processed several times using the EMD algorithm until the first IMF component is generated. The second IMF component is expressed as:
  (3) $$IM{F^{\prime }}_{2}\left(t\right)=\frac{1}{I}\sum_{i=1}^{I}E_{1}\left(r_{1}\left(t\right)+\varepsilon_{1}E_{1}\left(n^{i}\left(t\right)\right)\right)$$
(4) Perform step (3) above for the other remaining stages (j=2,3,⋅⋅⋅J), then the j+1 IMF component is expressed as:
  (4) $$r_{j}\left(t\right)=r_{j-1}-IMF_{j}\left(t\right)$$
  (5) $$IM{F^{\prime }}_{j+1}\left(t\right)=\frac{1}{I}\sum_{i=1}^{I}E_{1}\left(r_{j}\left(t\right)+\varepsilon_{j}E_{j}\left(n^{i}\left(t\right)\right)\right)$$
(5) Add 1 for *j* and repeat step (4) until the residual sequence cannot be processed. The number of IMF components is *J*. The final calculated residual sequence is expressed as:
  (6) $$r\left(t\right)=x\left(t\right)-\sum_{j=1}^{J}IMF_{j}{}^{\prime }\left(t\right)$$
(6) The original signal x(t) represented by the IMF component and the residual component is expressed as:
  (7) $$x\left(t\right)=\sum_{j=1}^{J}IMF_{j}^{\prime }\left(t\right)+r\left(t\right)$$

The CEEMDAN algorithm introduces a segment of positive and negative white noise for each stage processing, and overcomes the reconstruction error defect of the EEMD algorithm by processing only one residual component to find each IMF component. The CEEMDAN algorithm is able to reconstruct the decomposed signal with close to zero deviation. However, it also has shortcomings, for example, the definition of noise and useful signal in IMF components containing more noise is relatively ambiguous, and direct removal of these components can cause signal distortion.

#### 2.1.2. DFA Algorithm

To solve the problem of CEEMDAN for noise and useful signal demarcation ambiguity, the DFA algorithm is more accurate [33].

(1) $\bar{x}(i)$ is defined as the average of the time series x(i) in the time intervals [1,*N*], and denoted as:
  (8) $$\bar{x}=\frac{1}{N}\sum_{i=1}^{N}x\left(i\right)$$
  where *N* is the number of segments of length *n*.
(2) The time series *y*(*k*) is segmented into segments of length *n.* It is denoted as:
  (9) $$y\left(k\right)=\sum_{i=1}^{k}\left[x\left(i\right)-\bar{x}\right],k=1,2,3,\cdot \cdot \cdot$$
(3) The trend $y_{s}\left(i\right)$ of each series segment is calculated as:
  (10) $$y_{s}\left(i\right)=\sum_{n=0}^{k}a_{n}i^{n}$$
(4) After removing the uncertain trend in each series segment, the second-order fluctuation coefficient of the segment series is expressed as:
  (11) $$F^{2}\left(n,s\right)=\frac{1}{n}\sum_{i=1}^{n}{\left(y\left[\left(s-1\right)n+i\right]-y_{s}\left(i\right)\right)}^{2}$$
  (12) $$F_{q}\left(n\right)={\left[\frac{1}{N_{n}}\sum_{s=1}^{N_{s}}F^{2}\left(n,s\right)\right]}^{1/2}$$
(5) Change the segment length *n* in step (1), and repeat steps (2) and (3) to obtain the change in the fluctuation function of the time series. The correlation of the time series represented by the Hurst function is expressed as:
  (13) $$H=\frac{\log_{2}F_{q}\left(n\right)}{\log_{2}\left(n\right)}=\frac{\log_{2}\left[\frac{1}{N_{n}}\sum_{s=1}^{N_{n}}F^{2}\left(n,s\right)\right]}{\log_{2}\left(n\right)}$$
(6) The relationship between the scalar function *α* and the time series fluctuation function *F*(*n*) is expressed as:
  (14) $$F\left(n\right)\propto n^{\alpha }$$

It can be seen that *α* is related to F(n), and F(n) is related to *H*. Therefore, *α* is related to H. The value of *α* is proportional to the smoothness of the signal. When 0 < *α* < 0.5, it indicates that the proportion of noise in the signal is very large; *α* = 0.5 indicates the signal is not correlated; the signal has a large correlation when *α* > 0.5 [34]. By comparing the values of *α* in the IMF components, the noise-dominated IMF components and the useful information-dominated IMF components can be distinguished.

DFA is an algorithm for correlation discrimination of non-smooth signals. It is used to confirm the critical value of the noise component and the useful signal component, which can guarantee the accuracy of signal correlation discrimination to a certain extent.

#### 2.1.3. Improved Wavelet Threshold Function

To solve the distortion problem of the signal caused by the CEEMDAN, a wavelet threshold function is introduced to denoise the dominant component of the noise. Traditional threshold functions include hard threshold functions and soft threshold functions. The hard threshold function can maintain the signal characteristics well, but there is a discontinuity at the set threshold *λ*, which will cause serious oscillations when the signal is reconstructed. The soft threshold function does not have the discontinuity at the set threshold, but the wavelet coefficients after threshold quantization will have a constant deviation, which leads to a large deviation for the signal characteristics. Therefore, Based on the advantages of the hard thresholding function and soft thresholding function, an improved wavelet threshold function is presented to reduce reconstruction error.

The improved threshold function must meet the following conditions.

(1) A good continuity is maintained at the set threshold;
(2) The threshold function has monotonicity and continuity when the wavelet coefficient is greater than the set threshold;
(3) The threshold function should have an asymptote, and the curve $y\left(x\right)=x$ can overlap with the asymptote.

The improved wavelet threshold function is expressed as:

(15) $${\hat{\Psi }}_{j,k}=\left\{ \begin{array}{ll} \mathrm{sign}\left(\Psi_{j,k}\right){\left[{\left|\Psi_{j,k}\right|}^{2}-{\left(\lambda e^{-\left(\left|\Psi_{j,k}\right|-\lambda \right)/k}\right)}^{2}\right]}^{1/2} & \left|\Psi_{j,k}\right|\geq \lambda \\ a\Psi_{j,k} & \left|\Psi_{j,k}\right|<\lambda \end{array}\right.$$

where *k* and ɑ in the expression are adjustable, *k* > 0, a∈(0.05,0.5).

The characteristics of the improved wavelet threshold function are as follows:

(1) When $\psi_{j,k}\to \lambda$, $e^{-\left(\left|\psi_{j,k}\right|-\lambda \right)/k}\to 1$ and ${\overset{⌢}{\psi }}_{j,k}\to 0$, the improved wavelet threshold function is continuous at the threshold λ. When $\psi_{j,k}\to \infty$, $e^{-\left(\left|\psi_{j,k}\right|-\lambda \right)/k}\to 0$ and ${\overset{⌢}{\psi }}_{j,k}\to \psi_{j,k}$, the improved wavelet threshold function is an asymptote, which makes the reconstructed signal closer to the actual value.
(2) When k→0, the properties of the improved wavelet threshold function are close to the hard threshold function. When k→∞, the properties of the improved wavelet threshold function are close to the soft threshold function.
(3) When $\left|\psi_{j,k}\right|\leq \lambda$, a very large part of the wavelet coefficient is noise, and the *a* value is adjusted to be as small as possible to remove the noise interference.

The selection of the threshold value has a large impact on the signal denoising effect. The formula for the threshold selection rule used in this paper is expressed as:

(16) $$\lambda_{i}=\frac{\delta \sqrt{2\ln\left(N\right)}}{\ln\left(i+1\right)}$$

where δ is for noise intensity. *i* is the number of decomposition layers. *N* is the signal length. The threshold $\lambda_{i}$ varies with the number of decomposition layers. The wavelet coefficients of a noisy signal are inversely related to the number of decomposition layers. To ensure that the reconstruction error of the signal after denoising is small, the threshold $\lambda_{i}$ should be dynamically smaller as the number of decomposition layers increases.

### 2.2. The Validation of CEEMDAN-DFA-Improved Wavelet Threshold Function Denoising Algorithm

**Figure 1:**
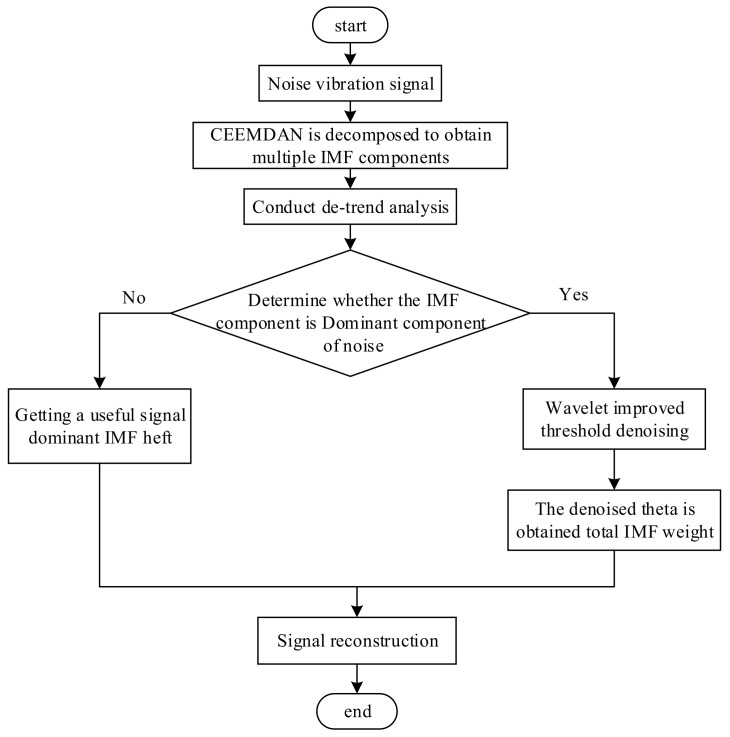
The operational flow of the CEEMDAN-DFA-improved wavelet threshold function algorithm.

The operational flow of the CEEMDAN-DFA-improved wavelet threshold function denoising algorithm is shown in Figure 1. Firstly, the CEEMDAN is used to decompose the vibration signal of the rolling bearing to obtain a series of IMF components. Then, the IMF is analyzed by DFA, and the scaling function values of each order of IMF are calculated. According to the correlation of DFA, the IMF component dominated by noise is selected, and the improved wavelet threshold function is used for denoising. Finally, the IMF component after denoising and the IMF component dominated by useful signals are reconstructed, and the reconstructed signal is the bearing vibration signal after denoising.

**Figure 2:**
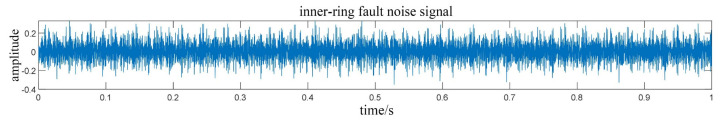
The time domain waveform of the inner-ring fault noise signal.

The verification data of the denoising algorithm come from the rolling bearing database provided by the Electrical Engineering Laboratory of Case Western Reserve University (CWRU) [35]. The validation data selected for this paper were the inner rings of a bearing with a damaging size of a 0.007-inch signal. To meet match the actual working environment, random Gaussian white noise was superimposed on the used bearing inner-ring fault signal. Figure 2 shows the time domain waveform of the bearing inner-ring fault noise-containing signal. The noise-containing signal was first decomposed using CEEMDAN to obtain the 12th-order IMF component, and then the IMF was calculated by the DFA for the scaling function value α. The calculation results are shown in Table 1. From the table, it can be seen that the scaling function value α of the first four-order IMF components was less than 0.5. Therefore, it can be determined that the main component of the first fourth-order IFM component is noise, and the main component of the remaining IMF is useful information. Signal reconstruction was performed on the first fourth-order IMF, and the reconstructed signal was denoised.

**Figure 3:**
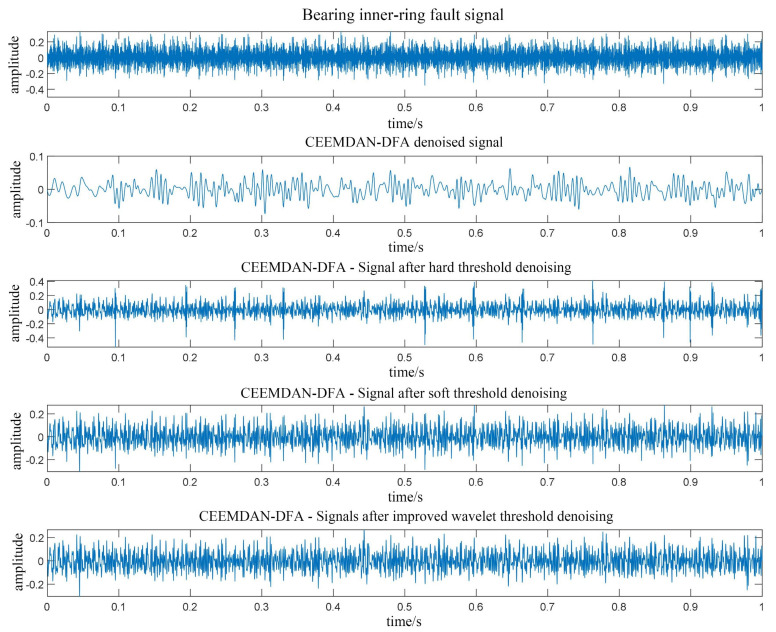
The time domain waveform of inner-ring fault noise signal.

To compare the advantages of the proposed denoising algorithms, four denoising methods were used, including CEEMDAN-DFA, CEEMDAN-DFA-hard thresholding, CEEMDAN-DFA-soft thresholding and the CEEMDAN-DFA-improved wavelet thresholding. The denoising results are shown in Figure 3.

The results show that both CEEMDAN-DFA and CEEMDAN-DFA-hard threshold denoising methods cause large signal distortion. The values of the signal-to-noise ratio (SNR) and root-mean-square error (RMSE) using the four denoising methods to the inner ring fault signal are shown in Table 2. The proposed CEEMDAN-DFA-improved wavelet threshold denoising method improved the SNR by 11.4% and reduced the RMSE by 16.2% compared with the CEEMDAN-DFA-wavelet soft threshold denoising method. It can be seen that the proposed denoising method in this paper has better performance than other denoising methods.

## 3. Fault Feature Extraction and Identification Algorithm of the QPSO-MPE-SVM

### 3.1. Basic Algorithm Related to the QPSO-MPE-SVM

#### 3.1.1. MPE Algorithm

The theoretical foundation of MPE is based on multiscale analysis and alignment entropy. MPE was used to coarsely granulate the initial time series to create a multiscale time series [35]. The calculation procedure is described as follows:

(1) The initial time series $x\left(i\right)$ is coarsely granularized to obtain the coarse-grained series $y_{j}^{\tau }$, which is calculated as follow:
  (17) $$y_{j}^{\tau }=\frac{1}{\tau }\sum_{i=(j-1)+1}^{\tau }x_{i}\;1\leq j\leq \frac{N}{\tau }$$
(2) Calculate multi-scale alignment entropy based on sequence $y_{j}^{\tau }$.
  (18) $$MPE(x,\tau ,m,\lambda )=PE(y_{j}^{\tau },m,\lambda )$$

The MPE calculation requires the signal length *N*, delay time λ, embedding dimension *m*, and scale factor τ. The MPE can respond very well to the changes of vibration signals, but if the parameters are not selected properly, the permutation entropy values of multiple vibration signals will be overlapped, and thus the fault characteristics cannot be extracted [36].

#### 3.1.2. QPSO Algorithm

In order to achieve complete separation of the MPE values for different operating conditions of the bearings, the QPSO is introduced in this paper to optimize the initial parameters of the MPE. To find the global optimal solution, the calculation formula is expressed as [37]:

(19) $$m_{best}=\frac{1}{M}\sum_{j=1}^{M}P_{j}$$

(20) $$P=rP_{j}+\left(1-r\right)P_{g}$$

(21) $$L_{j}\left(t_{1}+1\right)=P\pm \varsigma \left|m_{best}-L_{j}\left(t_{1}\right)\right|\ln1/u$$

where *m_best_* is particle optimum average. *M* is race number. *P_g_* is the global optimal solution for the particle. ς is compression expansion factor.

The fitness function has a large impact on the optimization results of QPSO when studying the trend of the data.

The MPE values of time series $x\left(i\right)$ are a formed sequence $H_{p}\left(X\right)$, $H_{p}\left(X\right)$ is described as follows:

(22) $$H_{P}\left(X\right)=\left\{H_{P}\left(1\right),H_{P}\left(2\right),\cdot \cdot \cdot ,H_{P}\left(n\right)\right\}$$

Skewness formula is described as follows:

(23) $$ske={\frac{E\left[H_{P}\left(X\right)-H_{P}^{m}\left(X\right)\right]}{{\left[H_{P}^{d}\left(X\right)\right]}^{3}}}^{3}$$

where E is the expected value. $H_{p}^{m}\left(X\right)$ and $H_{p}^{d}\left(X\right)$ are the mean and standard deviations of $H_{p}\left(X\right)$.

The fitness function about QPSO is described as follows:

(24) $$F\left(X\right)=\frac{1}{ske^{2}+1}$$

The fitness function has a large impact on the optimization results of QPSO. The skewness value is inversely proportional to the fitness function. Therefore, by calculating the minimum value of the skewness, we can obtain the best fitness function value. Using this value to optimize the parameters of the alignment entropy, we can make the distinction between the alignment entropy values of different fault types more obvious and make it easier to carry out fault diagnosis and classification.

#### 3.1.3. SVM Algorithm

Assume that $\left\{\left(x,y\right)|i=1,2,\cdot \cdot \cdot ,k\right\}$ is the input training set, where *k* is the number of training set samples.$x_{i}\in T^{d}$ is the d-dimensional feature vector. $y_{i}\in \left\{-1,1\right\}$ denotes the category of sample $x_{i}$.

In order to maximize the sample interval, the optimization formula was constructed and expressed as follows [38]:

(25) $$\begin{array}{l} \underset{w,b}{\min}\frac{||w|{|}^{2}}{2}+C\sum_{i=1}^{k}\xi_{i}\\ s.t.\;y_{i}\left[\left(w_{i}x\right)+b\right]\geq 1-\xi_{i\;}i=1,2,\cdot \cdot \cdot ,k \end{array}$$

where *w* is the weighting of the optimal classification surface. *C* denotes the penalty parameter of the deviation item $\xi_{i}$. When C>0, $\xi_{i}$ is the error caused when the sample points are misclassified.

The above nonlinear planning problem was transformed into a linear planning problem using the Lagrangian equation. The Lagrangian equation fuses the objective function with the constraint function and then finds the optimum value of that equation, which is the optimal classification surface. The classification decision function was obtained as:

(26) $$f\left(x\right)=\mathrm{sgn}\left(\sum_{i=1}^{k}\alpha_{i}y_{i}K\left(x_{i},x\right)+b^{*}\right)$$

where $\alpha_{i}$ is a Lagrangian multiplier. $K\left(x_{i},x\right)$ represents the kernel function of the Mercer condition, which is denoted as follows:

(27) $$K\left(x,y\right)=\exp\left(-\frac{||x-y|{|}^{2}}{2g^{2}}\right)$$

where *g* indicates the complexity of the subspace distribution of the sample data.

### 3.2. The Validation of the QPSO-MPE-SVM Algorithm

**Figure 4:**
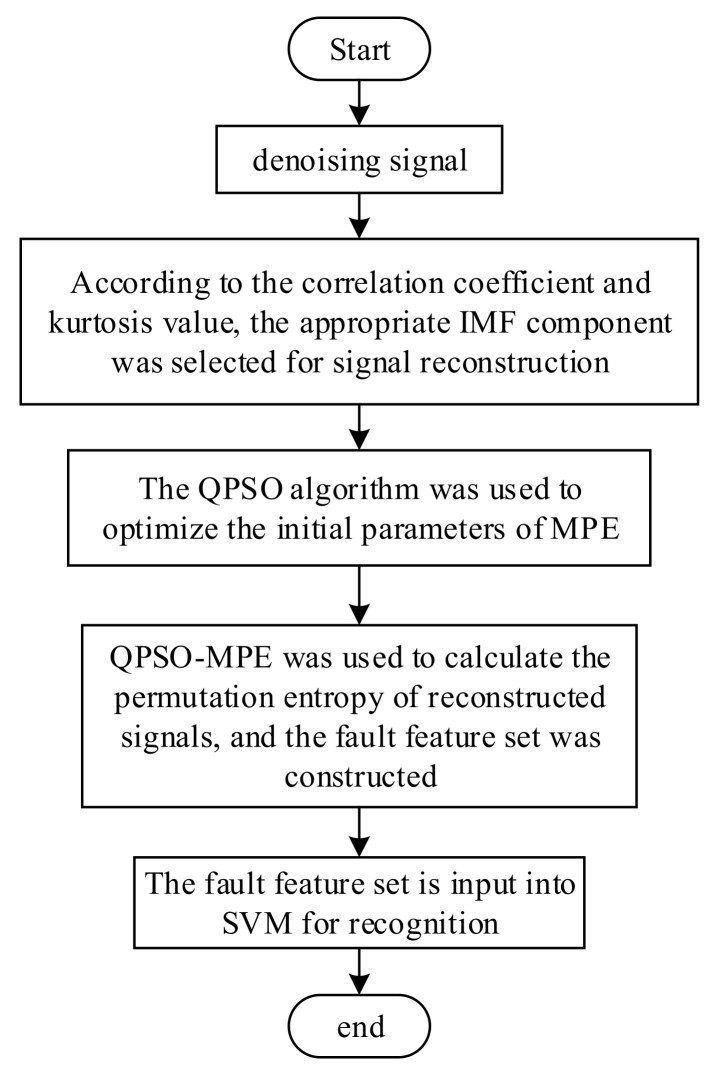
QPSO-MPE-SVM fault identification flow chart.

The process of QPSO-MPE-SVM fault feature extraction and identification is shown in Figure 4, which is as follows.

(1) The denoised vibration signal is again disintegrated by CEEMDAN, and the IMFs are selected according to the correlation coefficient and Kurtosis values of the IMF for signal reconstruction;
(2) The initial parameters of the MPE are optimized using the QPSO to obtain the better MPE parameters;
(3) The MPE values of the reconstructed signals are calculated using the optimized MPE parameters, and the MPE values with obvious differentiation are selected to construct the bearing fault feature set;
(4) The obtained MPE fault feature set is input to the SVM for fault identification.

The correlation coefficient value is proportional to the fault information content in the IMF component. The kurtosis value reflects the amount of shock information in the IMF component; the larger the kurtosis value, the more shock information in the IMF component, and the smaller the kurtosis value, the less oscillating information in the IMF component.

**Figure 5:**
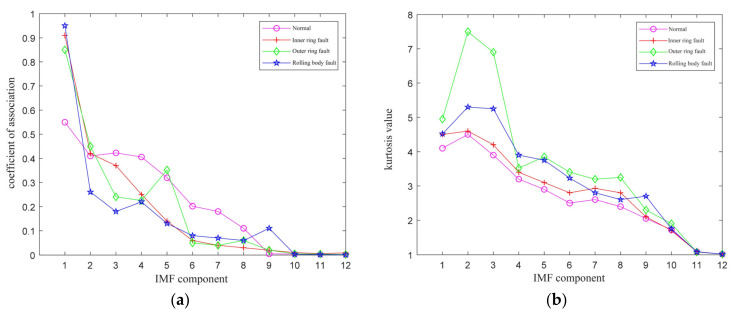
Plots of IMF component correlation coefficients and Kurtosis values. (**a**) The correlation coefficients of IMF; (**b**) The Kurtosis values of IMF.

The validated data came from the rolling bearing database provided by the Electrical Engineering Laboratory of Case Western Reserve University (CWRU) [35]. The correlation coefficient values and Kurtosis values of each order IMF component of the bearing fault signal (normal signal, inner-ring fault signal, outer-ring signal, and rolling-body fault signal) at 1797 rmp were calculated and the results are shown in Figure 5.

From Figure 5, it can be seen that the correlation coefficients of the four vibration signals were different, the correlation coefficients of the first five order IMFs of the four-fault signals were all above 10%, and Kurtosis values were all greater than 3. Therefore, the first fifth-order IMF components were selected for signal reconstruction.

**Figure 6:**
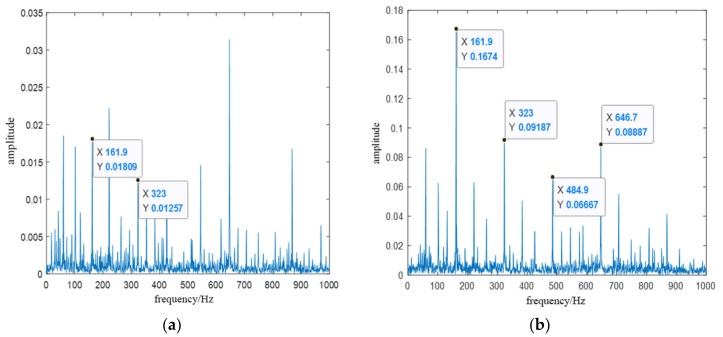
The HHT envelope spectrum of the inner-ring fault signal. (**a**) The envelope spectrum analysis of the original signal; (**b**) The envelope spectrum analysis of the reconstructed signal.

To prove whether the bearing fault characteristic information can be accurately extracted from the first fifth-order IMFs, we took the inner ring fault signal as an example for research. The CEEMDAN decomposed the fault signal of the bearing inner ring and selects the first-order and fifth-order IMF for signal reconstruction. Then, the reconstructed signal was analyzed by Hilbert-Huang transform (HHT) envelope spectrum. The envelope spectrum analysis of the original signal and the reconstructed signal is shown in Figure 6.

The theoretical value of the characteristic frequency of the inner-ring fault signal is 162.1 Hz, and the actual value is 161.9 Hz. The error of no more than 10% between the theoretical frequency value and the actual frequency can be determined as the same fault. From the original signal envelope spectrum shown in Figure 6a, it can be seen that there were other interference peaks near the peak of the fault characteristic frequency (161.9 Hz), which could not effectively extract the fault characteristic frequency. The HHT envelope spectrum of the reconstructed signal shown in Figure 6b, indicating that the characteristic frequency (161.9 Hz) can be easily extracted, and it is also easy to extract the di-frequency (323 Hz) and tri-frequency (484.9 Hz) of the fault features.

In summary, it was proved that the first fifth-order IMF components can retain the fault feature information, which further verifies the effectiveness of the proposed denoising method in this paper. This scheme can realize the real-time processing of fault signals and can be looped.

#### 3.2.1. Optimize MPE Values Using QPSO

**Figure 7:**
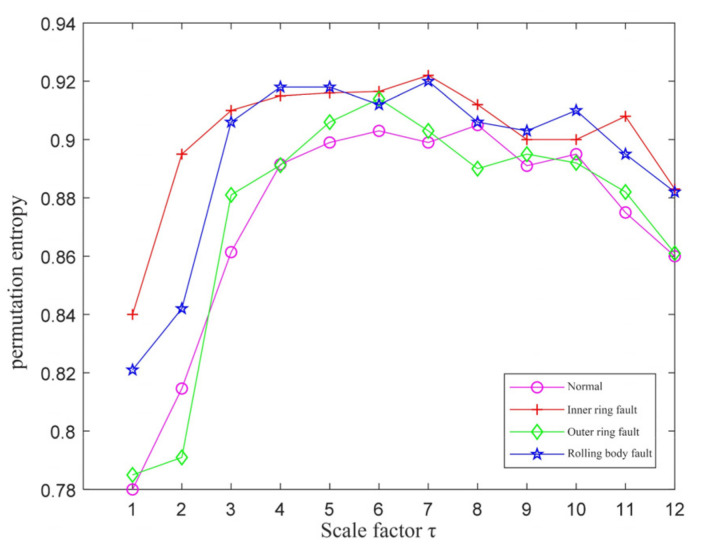
The MPE values without QPSO optimization.

The initial parameters of MPE were set to *N* = 1024, λ=1, m=6 and τ=12. The first fifth-order IMF components of the normal signal, inner-ring fault signal, outer-ring fault signal, and rolling-element fault signal were selected for signal reconstruction. The MPE values reconstructed signal was calculated and the results are shown in Figure 7. It can be seen that the MPE values of each signal were not distinguishable, so they could not be used as an effective feature set for fault classification.

**Figure 8:**
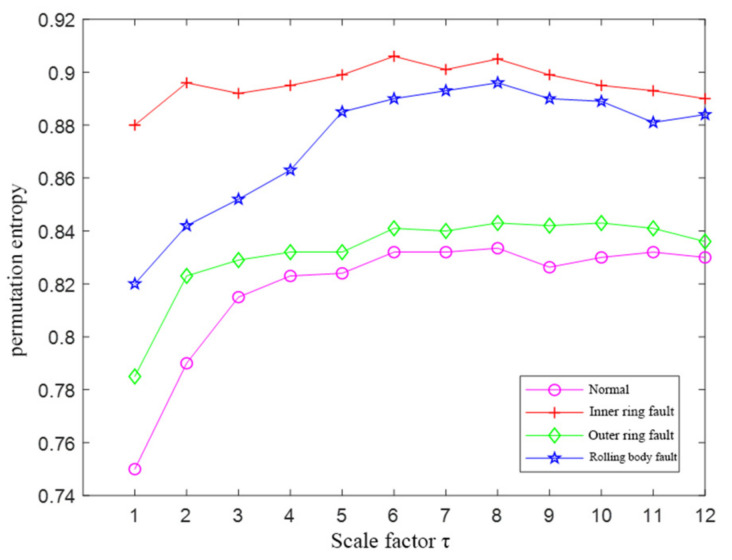
The MPE values optimized after QPSO.

To separate the MPE values of the four operating conditions of the bearings, the QPSO was used to optimize the initial MPE parameters. The parameters of QPSO were set to 30 for the number of races, 200 for the maximum number of iterations, and 10 and 0.2 for the maximum and minimum inertia weights, respectively. The parameters of the optimized MPE are shown in Table 3. The MPE values were calculated using the optimized parameters, and the results are shown in Figure 8. It can be seen that the MPE values of each signal were independent of each other, which can better construct the fault feature set. In this paper, the scale factors with stable and larger partition degrees were selected to construct the fault feature vector, so the selected factors were 5, 6, 7, 8, and 9.

#### 3.2.2. Fault Feature Extraction and Identification Using the QPSO-MPE-SVM

**Figure 9:**
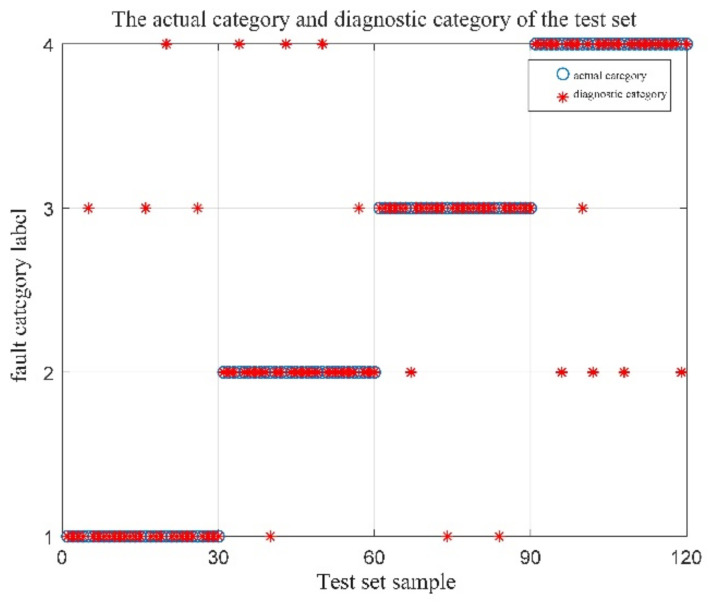
MPE-SVM fault identification results.

**Figure 10:**
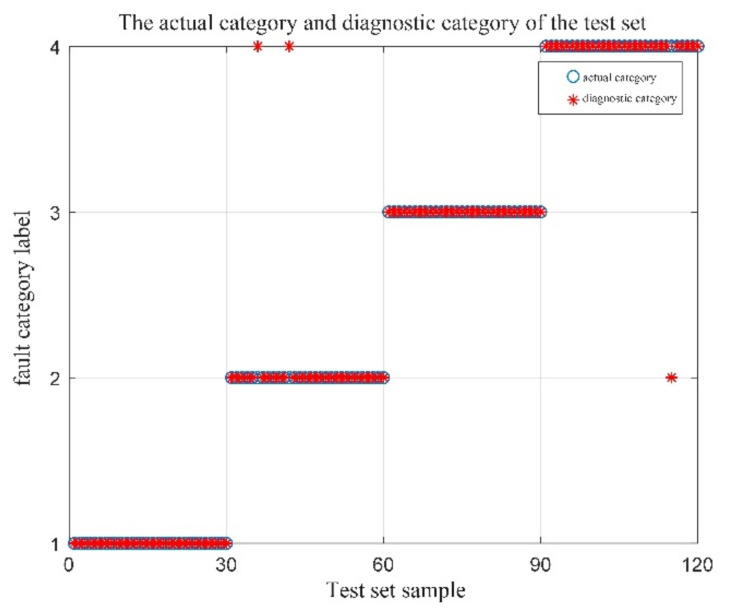
QPSO-MPE-SVM fault identification results.

Sixty sets of vibration data were selected for each of the four operating conditions of the bearings, for a total of 240 sets. These data were divided equally into two groups, one for the training set, and the other for the test set. Two fault-identification experiments were operated. One experiment is with the initial parameters of MPE, and the fault identification results are shown in Figure 9; the other experiment was with the optimized parameters of MPE by QPSO, and the fault identification results are shown in Figure 10. The indications in the figure include 1 for the normal state, 2 for the inner-ring failure state, 3 for the outer-ring failure state, and 4 for the rolling-element failure state.

Analysis of Figure 9 and Figure 10 reveals that when the MPE values without optimization were directly input to the SVM for fault identification, the fault identification accuracy was 85.83%, while the fault identification accuracy could reach 97.5% after optimization by the QPSO. In order to verify the reliability of the proposed method, several simulation experiments were repeated, and the simulation results all showed that the proposed method could effectively improve the accuracy of bearing fault diagnosis. Thus, the effectiveness of the QSPO-MPE-SVM method proposed is confirmed.

## 4. Fault Diagnosis of Rolling Bearing of Sine Roller Screen Based on Vibration Signal

**Figure 11:**
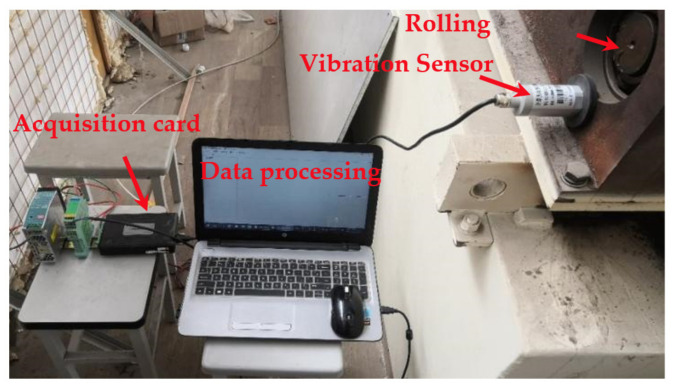
Sine roller screen rolling bearing fault experimental platform.

The experimental platform is shown in Figure 11. The experimental platform completes the tasks of installing sensors, driving bearings, applying radial loads and acquiring vibration signals. The test bearing was a grease-lubricated deep-groove ball bearing, and rolling bearing parameters as shown in Table 4. The working limit temperature of the bearing is 400 °C. The rotational speed is 10–800 r/min, and the limit rotational speed is 2000 r/min. The speed can be continuously adjusted, and the error is less than ±1%. The applied radial load is 120 kg, the ultimate is 500 kg, the model number of the load sensor is MZLF-2, and the sensor sensitivity is 2.0 ± 0.01 mV/V. The model number of the acceleration sensor is KH-HZD-B-2-12, and the sensitivity is 20 ± 0.05 mV/mm/s. The model number of data acquisition cards is USB3200N (32 bit, 4 channels, 20 MHz); the vibration sensor (MIC-HZB-F-2-12) collected the vibration acceleration of the bearing in real-time, and transferred the vibration signal to the data-acquisition card. The signal was finally transferred to the computer for data processing. All the experimental data were within the allowable error range.

### 4.1. Rolling Bearing Feature Frequency Calculation

The rolling bearing speed is 500 r/min. The theoretical formula of the fault frequency is expressed as:

(1) Journal rotation frequency is expressed as:

(28) $$f_{r}=\frac{v}{60}$$

(2) Inner-ring fault characteristic frequency is expressed as:

(29) $$f_{a}=\frac{nf_{r}}{2}\left(1+\frac{d}{D}\cos\theta \right)$$

(3) Outer-ring fault characteristic frequency is expressed as:

(30) $$f_{b}=\frac{nf_{r}}{2}\left(1-\frac{d}{D}\cos\theta \right)$$

(4) Rolling-body fault characteristic frequency is expressed as:

(31) $$f_{d}=\frac{Df_{r}}{2d}\left[1-{\left(\frac{d}{D}\right)}^{2}\cos^{2}\theta \right]$$

where *v* is journal rotation speed.

The calculation results by the formulas are shown in Table 5.

### 4.2. Rolling Bearing Fault Signal Denoising and Feature Extraction

**Figure 12:**
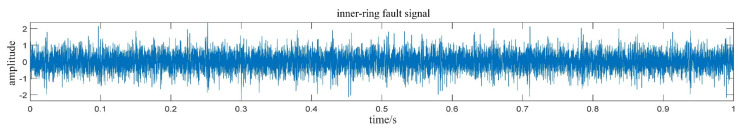
Time domain diagram of the original signal of the inner-ring fault.

**Figure 13:**
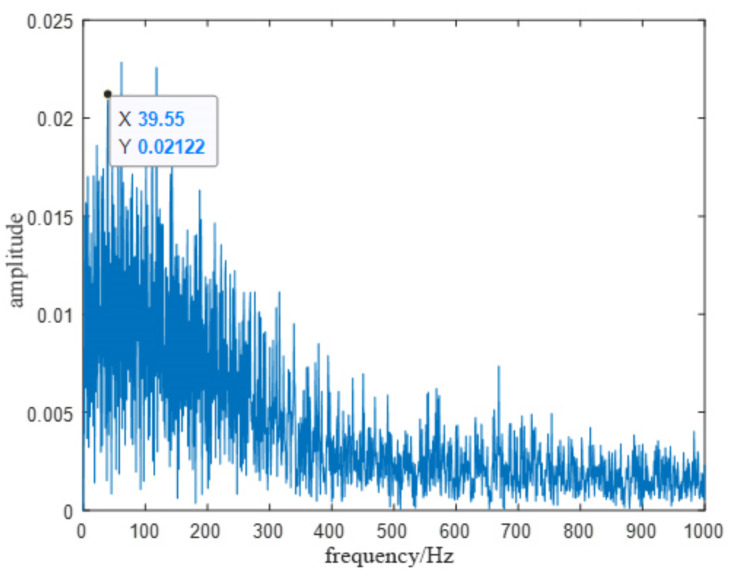
Original signal envelope spectrum of the inner-ring fault.

Taking the inner ring fault signal as an example, the original vibration signal and HHT envelope spectrum of the inner-ring fault are shown in Figure 12 and Figure 13, respectively. It was denoised using CEEMDAN-DFA-improved wavelet thresholding.

The IMFs were generated by the CEEMDAN decomposition, and the value of the scaling function α of the IMFs was calculated. α values of the first eighth-order IMFs are shown in Table 6.

**Figure 14:**
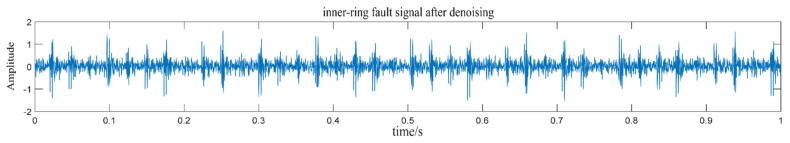
Time domain diagram of the inner-ring fault signal after denoising.

According to the DFA, correlation can be judged that the first fourth-order IMFs were dominated by noise, so the first four-order IMFs need to be denoised. The time-domain signal of the inner-ring fault after denoising is shown in Figure 14. The denoised signal and the remaining IMFs were reconstructed, and the reconstructed signal is the fault signal of the bearing inner ring.

The denoised signal was again decomposed by the CEEMDAN, and the correlation coefficients and Kurtosis values of each IMF were calculated. The calculation results are shown in Table 7.

**Figure 15:**
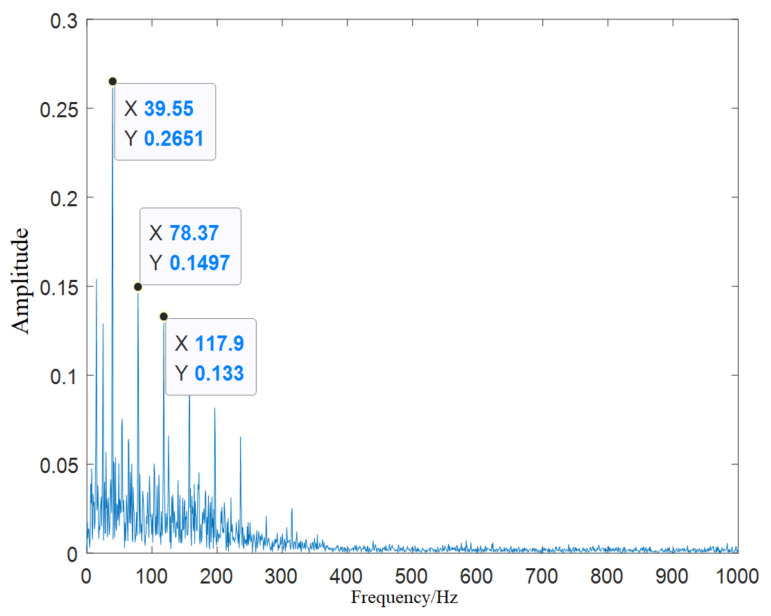
Envelope spectrum of the inner-ring fault signal after denoising.

From Table 7, it can be found that the correlation coefficients of the first fifth-order IMF components were greater than 0.1, and the Kurtosis values were greater than three. Therefore, the first fifth-order IMF component signal was reconstructed, and the HHT envelope spectrum of the reconstructed signal was analyzed. The analysis result is shown in Figure 15. It can be seen that the characteristic frequency of the inner-ring fault signal was 39.55 Hz, which is close to the theoretically calculated value (40.143 Hz).

**Figure 16:**
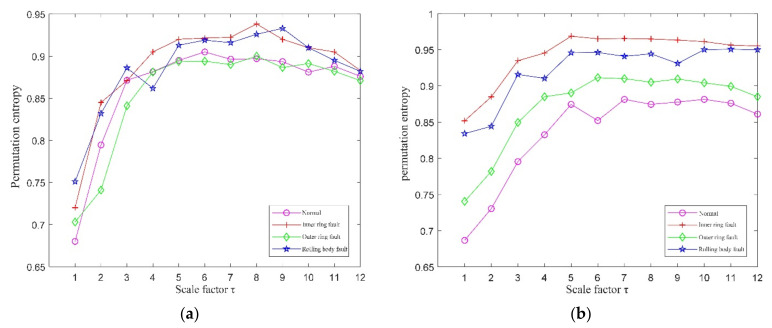
The MPE values of the four running signals. (**a**) The MPE values using the initial parameters; (**b**) The MPE values using the optimized parameters.

The initial parameters of the MPE of the four running signals were all set as N = 1024, λ=1, m=6, τ=12. The MPE values were calculated using the initial parameters, and the results are shown in Figure 16a. It can be seen that the MPE values of four signals had small differences and overlapped each other, which is not conducive to the construction of the fault feature set.

The initial parameters were optimized using the QPSO, and the results are shown in Table 8.

The MPE values of the four running signals were calculated using the optimized parameters, and the results are shown in Figure 16b. It can be seen that the MPE values of the four signals achieved separation without overlap, which is suitable for constructing fault feature sets for fault-type identification. The MPE values with scale factors (5, 6, 7, 8, 9, and 10) were selected to construct the fault feature vectors.

### 4.3. Rolling Bearing Failure Identification and Control Experiment

**Figure 17:**
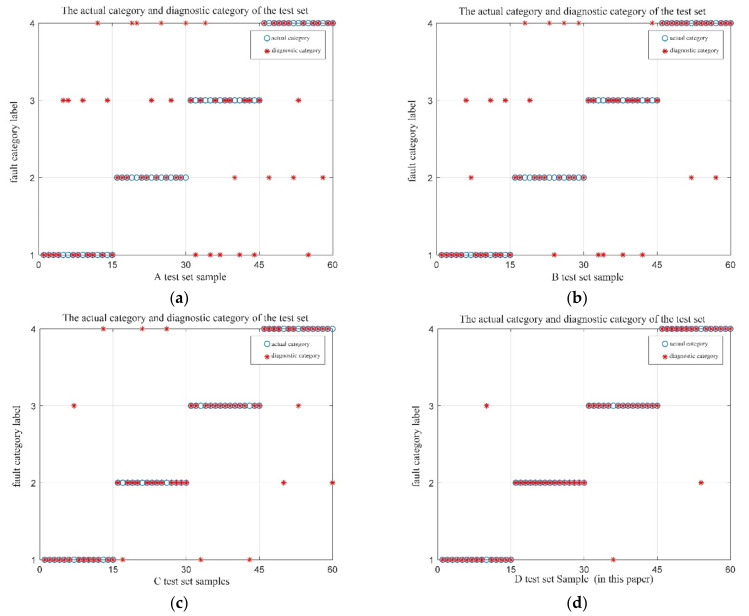
Four types of test sets identify results. (**a**) The results of MPE-SVM fault identification on the original signal; (**b**) the results of the QPSO-MPE-SVM fault identification on the original signal; (**c**) the results of MPE-SVM fault identification on the denoised signal; (**d**) the results of QPSO-MPE-SVM fault identification on the denoised signal.

A total of 160 sets of vibration experimental data were selected from 40 sets of four running signals, of which 100 sets were used as training samples and the remaining 60 sets were used as test samples. Comparing the four test sets, the recognition results are shown in Figure 17. The fault identification result of the original signal by MPE-SVM is shown in Figure 17a. The fault identification result of QPSO-MPE-SVM to the original signal is shown in Figure 17b. The results of MPE-SVM fault identification on the denoised (CEEMDAN-DFA-improved wavelet threshold function) signal are shown in Figure 17c. The results of QPSO-MPE-SVM fault identification on the denoised (CEEMDAN-DFA-improved wavelet threshold function) signal are shown in Figure 17d.

The following conclusions can be drawn from the analysis of Figure 17.

When using the MPE-SVM method for fault identification on the original signal without denoising, there were 23 false identifications, and the identification accuracy was only 61.67%. When using the QPSO-MPE-SVM method for fault identification on the original signal without denoising, there were 17 false identifications, and the identification accuracy was 71.67%, which shows that the noise in the collected vibration signal had a large interference to the bearing fault diagnosis.

When using the MPE-SVM method for fault identification on the denoised signal, the number of false identifications was reduced to 10, and the identification accuracy increased to 83.33%. When using the QPSO-MPE-SVM method for fault identification on the denoised signal, the number of false identifications was only three and the fault identification accuracy was 95%.

By applying the comprehensive diagnosis method proposed in this paper to the experimental platform of rolling bearings, the fault diagnosis of the actual measured fault signal of the inner ring of the bearing was achieved. The de-noising process and fault feature extraction were completed, and the fault feature set was constructed to achieve fault identification. The experimental results showed that the fault identification accuracy could be 95%. The method was verified, by several experiments, to not only have high accuracy and reliability, but also not be limited to inner-ring fault diagnosis, also being applicable to vibration signals with non-smooth and non-linear characteristics (such as outer-ring fault signals). Therefore, the method has good practical application value.

## 5. Conclusions

This paper proposed a CEEMDAN-DFA-improved wavelet threshold denoising method and QPSO-MPE-SVM fault feature extraction and identification method, the purpose being to realize the fault diagnosis of the rolling-bearing vibration signal.

The denoising process with the CEEMDAN-DFA-improved wavelet threshold method is as follows: Firstly, the vibration signal is decomposed into IMF by CEEMDAN, and the DFA is performed on the IMF components. Then, the scaling function of each IMF component is calculated to select the noise-dominated IMF component. Finally, the improved wavelet threshold function is applied to denoise the noise-dominated IMFs. The de-noised IMFs and the remaining other IMFs are merged to get reconfigured signals. The validation results showed that compared with traditional denoising methods, the CEEMDAN-DFA-improved wavelet threshold function method proposed in this paper could better remove noise, effectively reduce the signal distortion, and maintain the original characteristics of the signal.

The fault feature extraction and identification with the QPSO-MPE-SVM method are as follows: Firstly, the reconfigured signal is decomposed again by CEEMDAN, and the correlation coefficients and Kurtosis values of each order IMF component are calculated; the IMF components with larger values about correlation coefficients and Kurtosis are selected for signal reconstruction, and the HHT envelope spectrum analysis is performed on the reconstructed signal to extract the fault characteristic frequencies. Then, the initial parameters of MPE are optimized with QPSO, the MPE value is calculated for the reconstructed signal, and the appropriate MPE value is selected to construct the rolling-bearing fault feature vectors. Finally, the fault feature vectors are inputted to the trained SVM for rolling-bearing fault type identification. The validation results showed that the MPE parameters were optimized by the QPSO, which makes the MPE values of the four signals achieve separation without overlap, which is more suitable for constructing fault feature sets for fault type identification.

The algorithms proposed in the paper were all validated by building an experimental platform of rolling bearings. The experimental results showed that the fault identification accuracy of rolling bearings could reach 95%; it not only has high accuracy and reliability, but also can be applicable to vibration signals with non-smooth and non-linear characteristics, having good practical application value.

## Figures and Tables

**Table 1:** Scalar function values of each order IMF of the bearing inner ring fault signal.

**IMF**	**1**	**2**	**3**	**4**	**5**	**6**
*α*	0.4639	0.4214	0.3651	0.3124	0.5154	0.5712
**IMF**	**7**	**8**	**9**	**10**	**11**	**12**
*α*	0.6812	0.7948	0.8106	0.8637	0.8961	0.8942

**Table 2:** The SNR and RMSE of the inner-ring fault denoising.

Method	SNR	RMSE
CEEMDAN-DFA	4.9134	0.12143
CEEMDAN-DFA-wavelet hard threshold function	9.7542	0.08719
CEEMDAN-DFA-wavelet soft threshold function	13.6718	0.06024
CEEMDAN-DFA-improved wavelet threshold function	15.2324	0.05047

**Table 3:** Parameters of MPE after QPSO optimization.

Signal	N	λ	m	τ
Normal signal	1182	1	5	12
Inner-ring fault signal	1467	2	6	14
Outer-ring fault signal	1384	3	7	13
Rolling-body fault signal	953	1	6	12

**Table 4:** Rolling bearing parameters.

$\mathrm{Inner}\;\mathrm{Ring}\;\mathrm{Diameter}\;D_{1}$	$\mathrm{Outer}\;\mathrm{Ring}\;\mathrm{Diameter}\;D_{2}$	Rolling Body Diameter d	Bearing Mid Diameter D	Number of Rolling Bodies n	Contact Angle θ
70 mm	130 mm	20.43 mm	100 mm	8	0°

**Table 5:** The fault feature frequency of rolling bearing.

Rotational Speed	Inner Ring Fault Frequency	Outer Ring Fault Frequency	Rolling Body Fault Frequency
500 r/min	40.143 Hz	26.523 Hz	19.545 Hz

**Table 6:** Scalar function value of each IMF component of the inner-ring signal.

IMF	IMF_1_	IMF_2_	IMF_3_	IMF_4_	IMF_5_	IMF_6_	IMF_7_	IMF_8_
α value	0.4138	0.4096	0.2961	0.4537	0.5068	0.5564	0.6224	0.6743

**Table 7:** Correlation coefficients and Kurtosis values of IMFs of the inner-ring signal.

IMF	IMF_1_	IMF_2_	IMF_3_	IMF_4_	IMF_5_	IMF_6_	IMF_7_	IMF_8_
Correlation coefficient	0.9214	0.4327	0.3961	0.2715	0.2049	0.0621	0.0592	0.0357
Kurtosis value	4.5217	4.9371	4.1964	3.4922	3.2614	2.5147	2.2291	2.0634

**Table 8:** The optimized parameters of MPE after QPSO.

Signal	*N*	*λ*	*m*	*τ*
Normal Signal	1285	1	5	12
Inner-ring fault signal	1836	2	7	14
Outer-ring fault signal	1587	1	6	14
Rolling-body fault signal	1054	1	5	13

## Data Availability

https://csegroups.case.edu/bearingdatacenter/pages/12k-drive-end-bearing-fault-data accessed on 30 August 2021.
